# Impact of unilateral and bilateral salpingectomy on ovarian reserve

**DOI:** 10.5935/1518-0557.20250004

**Published:** 2025

**Authors:** Mohamed Atef Behery, Eman Ahmed Ali, Khaled Esmail

**Affiliations:** 1 Professor of Obstetrics, Gynecology and Reproductive Medicine, ART Unit, Al-Azhar university, Cairo, Egypt; 2 Consultant of Obstetrics, Gynecology and Reproductive Medicine, ART Unit, Al-Azhar university, Cairo, Egypt; 3 Specialist of Obstetrics, Gynecology, Damanhur Teaching Hospital, Behira, Egypt

**Keywords:** tubal surgery, salpingectomy, ovarian reserve change, AMH, ovarian follicles

## Abstract

**Objective:**

Salpingectomy has been one of the most performed surgical procedures in gynecological practice worldwide. Common indications include ectopic pregnancy and salpingitis (for example symptomatic hydro or pyosalpinx). This rising trend in salpingectomy has been associated with a rising concern over its potentially damaging effect on ovarian reserve due to possible concomitant damage of ovarian blood supply given the proximity of tubal and ovarian arteries.

**Methods:**

This is a prospective cohort Study including eighty cases of infertile women with a previous unilateral or bilateral hydro salpinx and indicated for salpingectomy or tubal ligation attending the outpatient clinic at assisted reproduction unit, Al-Azhar University. The results were obtained in the period from February 2020 to December 2022.

**Results:**

The present study was conducted on 80 patients. The mean and (SD) values for age were 25.5 (5.2) years old with a minimum of 18 and a maximum of 34 years old. More than half of patients (58.8%) had bilateral Hydrosalpinx while 41.2% had unilateral hydrosalpinx. Whether preor post-operatively, there was no statistically significant difference between median number of antral follicles in patients with unilateral and bilateral hydrosalpinx. There was no statistically significant change in the mean of AMH levels post-operatively (*p*-value=0.147, effect size=0.035) in both unilateral and bilateral groups. There was no statistically significant change in median number of Antral follicles post-operatively (*p*-value=0.456, Effect size=0.167) in both unilateral and bilateral groups.

**Conclusions:**

The unilateral and bilateral salpingectomy in patients with normal ovaries has no detrimental effect on ovarian reserve.

## INTRODUCTION

Tubal infertility, which is the main indication of in vitro fertilization (IVF)-embryo transfer accounts for about 25% to 35% of female infertility. The most severe manifestation of women suffering from tubal disease is hydrosalpinx (Wu *et al.*, 2020).

This rising trend in salpingectomy has been associated with a rising concern over its potentially damaging effect on ovarian reserve due to possible concomitant damage of ovarian blood supply given the proximity of tubal and ovarian arteries. It has therefore been hypothesized that salpingectomy could interrupt ovarian blood supply, thereby compromising ovarian blood flow with a consequent decline in ovarian reserve (Kwon *et al.*, 2015). Currently, laparoscopic salpingectomy and tubal occlusion seem to be helpful to improve the success rate of IVF. Reproductive surgery is still necessary as a complementary treatment for optimizing IVF outcomes for patients with not only hydrosalpinges but also for select cases of endometriomas and myomas. In order to prevent signs or suspicions of hydrosalpinx, the National Institutes of Health and Clinical Excellence (NICE) has recommended laparoscopic salpingectomy before ART (NICE, 2017). As the anatomical position of the blood vessels and nerves supplying for the oviduct and ovary are close to each other, interruption of the blood supply of the ovary may occur after laparoscopic surgery, which lead to poor ovarian reserve (Wu *et al.*, 2020). As most women requiring salpingectomy are relatively young and still wishing to preserve their fertility potential, it will be critical to evaluate any possible impairment of their ovarian reserve. This will help both the clinician and the patient when considering the need for salpingectomy (Mohamed *et al.*, 2017). Although there are numerous markers for ovarian reserve, it is now universally agreed that circulating anti Mullerian hormone (AMH) is considered the most reliable test (Harrison *et al.*, 2014).

Several retrospective studies have shown that hydrosalpinx may significantly reduce the rate of embryo implantation and clinical pregnancy and increase the rate of abortion and ectopic pregnancy. The mechanism of hydrosalpinx affecting the success rate of IVF is still not completely clear. It is mainly believed that there are several reasons from the following aspects: hydrosalpinx fluid could return to the uterine cavity and may affect endometrial receptivity, cause an embryotoxic agent, mechanical hindrance to implantation and simply wash out embryos and so on. Thus, it is generally believed that patients with unilateral or bilateral hydrosalpinges would be better to have pretreatment of hydrosalpinx before their IVF treatment (Strandell & Lindhard, 2002). However, salpingectomy is a feasible, sure surgical procedure and an expert surgeon could minimize ovarian and tubal blood vessel injuries. Thus, it is worthwhile to search the actual benefits/harms of laparoscopic salpingectomy and tubal occlusion before IVF to ovarian reserve in women with hydrosalpinx. The main objective of this study was to assess and compare the ovarian reserve after salpingectomy.

## MATERIALS AND METHODS

### Study design

This is a prospective cohort Study including eighty cases of infertile women with a unilateral or bilateral hydro-salpinx and indicated for salpingectomy attending the outpatient clinic in assisted reproduction unit, Al-Azhar university. in the period from February 2020 to December 2022. Pre-operative estimation of ovarian reserve was conducted by anti-Mullerian hormone (AMH) and basal ultrasound to calculate the antral follicle count. Six months after surgery, the ovarian reserve was re-evaluated by the same markers to compare the changes in ovarian reserve after tubal surgery.

Inclusion criteria:

1. Infertile women with ages below 35 years.
2. Hydro-salpinx is indicated for salpingectomy.

Exclusion criteria:

1- Patients with suspected or known mullerian anomalies, ovarian pathology, poly cystic ovarian disease.
2- Systemic diseases like DM, chronic hypertension.
3- Previous ovarian or tubal surgery.

Consents were obtained from each participating woman. Sample size was calculated using Epi-info7 software for sample size calculation considering the power of the study to be 80%, the level of significance to be 5% and the effect size that gives the minimal clinical difference before and after the procedure to be 30.

### Operative procedure

Unilateral and bilateral salpingectomy was done for patients who were diagnosed with hydrosalpinx and complaining of infertility. Before the procedures, basal follicular count and AMH were evaluated to estimate the ovarian reserve (OR). The abdominal skin and umbilicus were cleaned with 10% povidone-iodine solution. Pneumoperitonization was achieved using a Veress needle inserted through the umbilicus and a 10-mm trocar was inserted at the same location for the optic system. Under direct visualization, 2 5-mm ancillary trocars were inserted into the left and right lower abdominal quadrants lateral to the inferior epigastric arteries. During laparoscopic salpingectomy, removal of the fallopian tubes was performed with either monopolar or bipolar electrosurgery based on the surgeon’s preference and the patient’s anatomy. Care was taken to avoid injury to the ovarian vessels and to divide the mesosalpinx as close to the fallopian tube as possible. Post-operative pain was managed with 1 g of intravenous paracetamol every 8 h to a maximum of 3 doses (the first dose was administered 15 min before skin closure). Rescue analgesia (1 g paracetamol) was administered on patient request. Six months after the procedure OR was re-evaluated using the same parameters (AMH, antral follicular count) which were used before surgery. The values of pre and post-operative ovarian reserve markers were compared to estimate if there is a change in ovarian reserve.

### Statistical Analysis

Numerical data were explored for normality by checking the distribution of data by using tests of normality (Kolmogorov-Smirnov and Shapiro-Wilk tests). All data had showed normal (parametric) distribution except for number of Antral follicles data which showed non-normal (non-parametric) distribution. Data were presented by mean, standard deviation (SD), median and range values. For parametric data, paired t-test, was used to evaluate the changes in different variables post-operatively. The student’s t-test was used to compare the markers estimated before and after unilateral and bilateral hydrosalpinx. For non-parametric data, Wilcoxon signed-rank test was used to evaluate the changes in number of antral follicles post-operatively. The Mann-Whitney U test was used to compare between unilateral and bilateral hydrosalpinx. The significance level was set at *p*≤0.05. Statistical analysis was performed with IBM SPSS Statistics for Windows, Version 23.0. Armonk, NY: IBM Corp.

### Ethical approval

The study was approved by the local ethical board at Al-Azhar University.

## RESULTS

**Figure 1:**
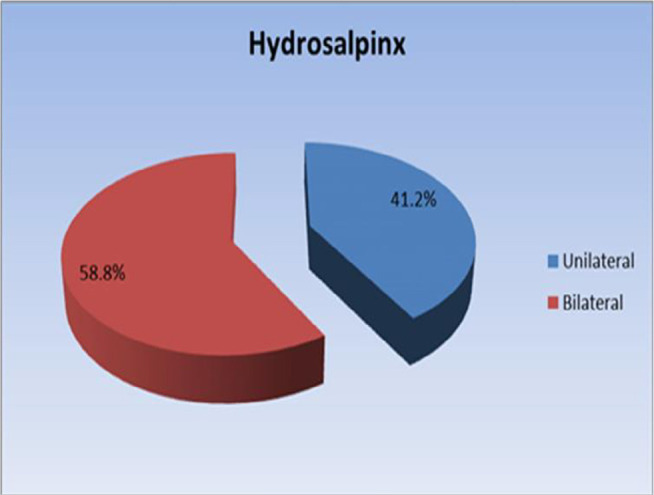
Pie chart representing distribution of hydrosalpinx in the study sample.

The present study was conducted on 80 patients. The mean and (SD) values for age were 25.5 (5.2) years old with a minimum of 18 and a maximum of 34 years old. More than half of patients (58.8%) had bilateral Hydrosalpinx while 41.2% had unilateral hydrosalpinx as shown in Figure 1.

**Figure 2:**
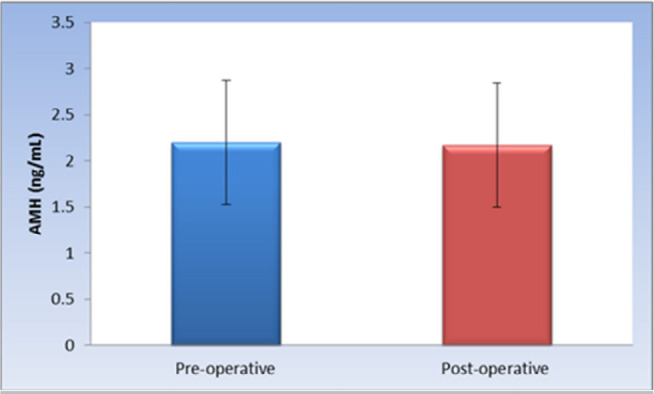
Bar chart representing mean and standard deviation values for AMH preand post-operatively.

There was no statistically significant change in the mean of AMH levels post-operatively (*p*-value=0.147, Effect size=0.035). The means and SDs are 2.2±0.67 and 2.17±0.67 with *p*=0.147 before and after salpingectomy respectively as shown in Figure 2.

The values of basal FSH showed non statistically significant difference in median number of antral follicles post-operatively (*p*-value=0.456, Effect size=0.167). The medians were 7.5 and 7 with *p*-value=0.456 for the group before and after the procedures, respectively. By the same comparison, there was no statistically significant change in mean FSH levels post-operatively (*p*-value=0.863, Effect size=0.009).

The means and SDs were (6.18±1.5 and 6.19±1.63 with *p*-value=0.863), respectively.

There was no statistically significant change in mean basal E2 levels post-operatively (*p*-value=0.150, Effect size=0.054). The means and SDs were (55.43±20.3 and 56.54±20.49 with *p*=0.150), respectively.

## DISCUSSION

Several retrospective studies have shown that hydrosalpinx may significantly reduce the rate of embryo implantation and clinical pregnancy and improve the rate of abortion and ectopic pregnancy (Wu *et al.*, 2020). The mechanism of hydrosalpinx affecting the success rate of IVF is still not completely clear. It is mainly believed that there are several reasons from the following aspects: hydrosalpinx fluid could return to the uterine cavity and may affect endometrial receptivity, cause an embryotoxic agent, mechanical hindrance to implantation and simply wash out embryos and so on. Thus, it is generally believed that patients with unilateral or bilateral hydro salpinges would be better to have pretreatment of hydrosalpinx before their IVF treatment (Vignarajan *et al.*, 2019).

It has been proved that salpingectomy performed as a pretreatment could significantly increase the rate of successful implantation and clinical and ongoing pregnancy (Chua *et al*., 2017). Nevertheless, both salpingectomy and tubal ligation for hydrosalpinx involve certain surgical risks. Ovarian blood supply mainly comes from the arterial arch in the ovarian artery and the mesosalpinx. Tubal excision may damage the arch of the artery, while tubal ligation at the proximal end and distal salpingostomy may cause less damage to the mesosalpinx (Ng & Cheong, 2019). Then the continuity of blood vessels between the oviduct and ovary is damaged, resulting in insufficient blood supply to the ovary and dysfunction, especially in women with previous abdominal surgery and/or extensive pelvic adhesions. Most of the surgery was completed at the 3rd-7th day after the end of menstruation. At this time, the antral follicles had formed already, and the effect may not come up immediately. In the second month after surgery, the new antral follicles were highly influenced by the effects of ischemia on the ovary sustained all the month and truly reflected the impact of the tubal surgery on ovarian reserve (Wu *et al*., 2020).

Whether salpingectomy affects ovarian function remains a controversial issue. Theoretically, since the median ovarian artery is very close to the medial tubal artery at their origins, an injudicious surgery to this area can undoubtedly jeopardize ovarian arterial supply which can in turn disrupt normal steroidal production and follicular development. An early study using 2D Doppler ultrasonography demonstrated an increase in the pulsatility index (PI) of the ovarian artery 3 months after Filshie clip sterilization (Chan *et al*., 2003). Surprisingly in two published meta-analyses that estimate the deleterious effects of hydrosalpinx on IVF/ICSI outcome, they concluded that surgery prior to ART consequently increases their success rate (Gizzo *et al*., 2015a,b).

The potential benefit of prophylactic laparoscopic salpingectomy before ART in case of hydrosalpinx were conducted. Data analysis in all trials showed a clear advantage in terms of implantation rate, pregnancy rate and ongoing pregnancy rate in treated patients compared to untreated controls. The derived data encouraged the scientific community to recommend tubal removal or tubal occlusion for hydrosalpinx prior to ART. Though these recommendations may be of use in the management of evident hydrosalpinx, controversy persists regarding the ideal management of smaller unilateral or bilateral hydrosalpinx due to the potential detrimental effects of surgery on the ovarian reserve (Noventa *et al.*, 2016).

The study included eighty patients who were diagnosed with moderate to severe, unilateral, or bilateral hydrosalpinx and attended a university assisted reproduction unit with a period of infertility and indicated for IVF/ICSI. The diagnosis of hydrosalpinx was confirmed by both pelvic ultrasound and hysterosalpingography. The results of current study demonstrate no detrimental effect on the ovarian reserve after laparoscopic salpingectomy either unilaterally or bilaterally as demonstrated by the non-change in ovarian reserve markers within the first 6 months after the procedure.

Many investigators recruited several relevant studies, regardless of study type. This approach allowed them to perform a thorough meta-analysis that included both a longitudinal/sequential comparison and case- control comparison. In addition, they focused on two reliable ovarian reserve markers: serum AMH levels and AFC. The decrease in ovarian reserve after salpingectomy is expected to be due to the reduction in ovarian blood flow caused by the incision of the mesosalpinx vascular network (Kobayashi *et al.*, 2022). As the previous studies, the current study included the two mostly accepted parameters for ovarian reserve evaluation, AMH and AFC in addition to FSH and E2 for more confirmation of any detrimental effect on the ovaries. However, it is well known nowadays that the latter two hormones are less sensitive than AMH and AFC in prediction of ovarian reserve.

In an interesting study by Page *et al.* (2021), they evaluated ovarian blood flow on the resected and healthy side by 3D power Doppler index in patients after laparoscopic salpingectomy for ectopic pregnancy. They reported that ovarian blood flow was decreased in the resected side. These results are totally different from our results, and this may be explained by the difference in indication of salpingectomy, the long-term effect of hydrosalpinx on the tubo-ovarian vascular plexus may lead to appearance of new vascular anastomosis around the tubes and ovaries that can compensate for the damaging effect on ovarian blood supply during salpingectomy. In contrary to salpingectomy for acute conditions like ectopic pregnancy, there is no time for neovascularization.

However, cystectomy for endometriomas with the detachment of the adhesions around the fallopian tube caused a decline of the AMH levels in the medium to long term in a study by Murase *et al.* (2019), they speculate that the damage to the mesosalpinx vascular network may be an independent reason for cystectomy itself. This may be attributed to direct damage to ovarian tissues during removal of the endometrioma and not due to vascular disturbance. These results were supported by a recent studyby Suneja *et al*. (2020) who found that the opportunistic salpingectomy did not affect ovarian reserve and vascularity at post 3 months of surgery; however, depletion of the ovarian reserve due to surgical intervention may be chronic; hence, follow-up assessment over a longer period may be required, especially in the younger patients.

Similarly, Wang & Gu (2021) found that prophylactic bilateral salpingectomy does not damage the ovarian function of reproductive-age women. With the same conclusion, Kobayashi *et al*. (2022) reported that there were no significant differences in the longitudinal evaluation of AMH levels before and after surgery, regardless of whether the case was unilateral or bilateral (Kotlyar *et al*., 2017; Zhu *et al*., 2021). However, the impact on IVF success and spontaneous pregnancy rates must be weighed by the indication for possible salpingectomy. This concept is totally in agreement with Kotlyar *et al.* (2017), who reported that, in patients planning for IVF, salpingectomy does not appear to significantly affect ovarian stimulation parameters or clinical pregnancy rates and has no significant effects on ovarian reserve (Suneja *et al.*, 2020).

Salpingectomy seems to have no short-term adverse effect on ovarian reserve as reported by many studies. However, given the possible concomitant damage to ovarian blood supply during salpingectomy, long-term adverse effect on ovarian reserve remains a concern that requires further investigations. In this study we investigate the mid-term effects of salpingectomy on ovarian reserve. Recently it was known that the effect of salpingectomy on ovarian reserve as well as ovarian response to controlled ovarian stimulation during IVF has not been proven. However, most of the studies recommended for prospective, as well as larger studies to confirm these results.

In non-fertile patients, one study by Kotlyar *et al*. (2017), was conducted to investigate short-term adverse effects of salpingectomy done at the time of hysterectomy for benign indications with preservation of ovaries. The study showed no effect on ovarian reserve and function (Kotlyar *et al*., 2017). However, this study had omitted the effect of age on ovarian reserve as well as the change in blood supply of pelvic organs in old women.

In contrary to these results, Ates *et al.* (2022) suggested that in women aged 35 to 39 years, salpingectomy may significantly decrease AFC, which may indicate declined ovarian reserve. Moreover, basal FSH, LH and E2 levels, total gonadotrophin doses, duration of gonadotrophin, fertilization rates, numbers of available embryos, and other pregnancy outcomes were similar between the salpingectomy group and the control group (Chen *et al.*, 2020). Begum *et al.* (2021) reported that salpingectomy in ectopic pregnancy may impair ovarian function and if there is bilateral salpingectomy it may shorten the reproductive life. Ovarian vascularization is provided by the ovarian and uterine arteries and is close to the fallopian tube and mesosalpinx. Thus, fallopian tube surgery may impair ovarian vascularization and function. The principle aim of the previous study was to evaluate the sonographic impact on the homolateral or bilateral ovarian response after salpingectomy for ectopic pregnancy (EP) during IVF stimulation. The Long-term effects of unilateral and bilateral salpingectomy need to be evaluated. Moreover, care should be taken during surgery to avoid accidental involvement of the part of the infundibulo-pelvic ligament during tubal clamping.

In the current study, we may not be able to conclude the detrimental effects of salpingectomy on ovarian reserve, although salpingectomy, especially bilateral surgery, may decrease the ovarian reserve evaluated with AMH and AFC as reported in the above-mentioned studies. Future research is needed to evaluate the sequential changes in AMH and/or AFC in the medium to long term after surgery for each indication and to determine the effect of different methods of salpingectomy (surgical techniques and energy devices) on ovarian reserve surgical techniques and energy devices) on ovarian reserve.

## CONCLUSION

Salpingectomy either unilateral or bilateral has no detrimental effect on ovarian reserve in the short-term follow up. However, the long-term effect on ovarian reserve remains uncertain.

### Limitations of the study

The study has points of strength that could be presented in being prospective with reasonable sample size. The weak points of the study are being observational and included the patients whose underwent unilateral and bilateral salpingectomy in the same cohort. This may limit clinical applicability of the results.
